# Hyperglycaemia in Pregnancy and Anthropometric Parameters in the Offspring at 10 Years: A Community-Based Retrospective Cohort Study in Sri Lanka

**DOI:** 10.1155/2020/2735148

**Published:** 2020-06-25

**Authors:** Himali P. Herath, Rasika P. Herath, Rajitha Wickremasinghe

**Affiliations:** ^1^Department of Nutrition, Medical Research Institute, Colombo 00800, Sri Lanka; ^2^Department of Obstetrics and Gynaecology, Faculty of Medicine, University of Kelaniya, Ragama, Sri Lanka; ^3^Department of Public Health, Faculty of Medicine, University of Kelaniya, Ragama, Sri Lanka

## Abstract

**Background:**

Studies of developmental origins of health and disease have highlighted the possible role of intrauterine hyperglycaemia, increasing the future risk of obesity, diabetes, and cardiovascular diseases in the offspring. There is limited evidence from South Asian populations for risk estimates for childhood obesity that are attributable to maternal diabetes in utero.

**Objective:**

The aim of this study was to determine the association between hyperglycaemia in pregnancy (HIP) and anthropometric parameters in the offspring at 10-11 years of age.

**Methods:**

A community-based retrospective cohort study was conducted in Colombo district, Sri Lanka. In the first stage, children born in 2005 were identified, and the availability of antenatal records was assessed. In the second stage, the exposure status of participants was ascertained based on antenatal records and predefined criteria. In the third stage, height, weight, waist circumference, and triceps skinfold thickness (TSFT) of eligible participants were measured to ascertain the outcome status. Background characteristics were collected by interviewing mothers. A 24-hour dietary recall and a 3-day diet diary were recorded.

**Results:**

159 children of mothers with HIP (exposed) and 253 children of mothers with no HIP (nonexposed) participated. Mean ages (SD) of exposed and unexposed groups were 10.9 (0.3) and 10.8 (0.3) years, respectively. The median BMI (17.6 vs 16.1, *p* < 0.001), waist circumference (63 cm vs 59.3 cm, *p* < 0.001), and triceps skinfold thickness (13.7 mm vs 11.2 mm, *p* < 0.001) were significantly higher in the exposed group. Offspring of women with HIP were more likely to be overweight (aOR = 2.6, 95% CI 1.4–4.9) and have abdominal obesity (aOR = 2.7, 95% CI 1.1–6.5) and high TSFT (aOR = 2.2, 95% CI 1.06–4.7) at 10-11 years than children who were not exposed after adjusting for maternal BMI, maternal age at delivery, and birth order.

**Conclusions:**

Intrauterine exposure to HIP is a significant determinant of overweight, high TSFT, and abdominal obesity in the offspring.

## 1. Introduction

Hyperglycaemia in pregnancy (HIP) is one of the most common medical conditions encountered in pregnancy. The International Diabetes Federation (IDF) estimates that one in six live births (16.2%) in the world and one in four live births (24%) in South East Asia are complicated with some form of hyperglycemia in pregnancy [1]. The number of women having hyperglycaemia in pregnancy is increasing as a result of the increasing prevalence of obesity and diabetes in women and higher age at childbirth [2].

Pederson's hyperglycemia-hyperinsulinism hypothesis, as supported by several studies, is still the basis of research on fetomaternal metabolism [3, 4]. This hypothesis postulates that deficiency of maternal insulin causes a rise in maternal glucose, which in turn increases fetal glucose levels. This results in fetal hyperinsulinaemia which stimulates fetal growth and adiposity. Frienkel and Metzger stated that deficiency of maternal insulin causes an increased influx of mixed nutrients or fuels (glucose, amino acids, lipids, and ketones) into fetal circulation resulting in hyperinsulinaemia [3]. Frienkel presented the concept “fuel-mediated teratogenesis” to describe alterations that goes beyond organogenesis causing long-range effects on anthropometric, metabolic, and behavioral functions in the offspring due to abnormal fuel mixtures in maternal metabolism due to hyperglycaemia [3]. Studies of developmental origins of health and disease have highlighted the possible role of hyperglycaemic intrauterine environment accelerating the current epidemic of obesity and diabetes through fetal programming and epigenetic changes [5, 6]. Metabolic changes in the intrauterine environment caused by different types of HIP appear to affect the growth of the developing foetus in a similar manner [7], and there is evidence that long-term consequences of intrauterine exposure to hyperglycaemia on the offspring are independent of mother's diabetes type [8, 9].

Several epidemiologic studies have investigated the association between HIP and offspring anthropometric outcomes during childhood in the past five decades, and the majority of earlier studies focused on Pima Indians and American birth cohorts. A large number of observational studies have reported a positive association between HIP and overweight and obesity in the offspring [10–19], but almost all of them were from the Western countries and America. Recent studies have investigated body composition measures beyond weight or BMI in the offspring of mothers with HIP. In the Hyperglycemia and Adverse Pregnancy Outcomes Follow-Up Study (HAPO FUS), offspring exposed to mild, untreated maternal hyperglycemia had increased adiposity, skinfold thickness, and waist circumference at a mean age of 11.4 years [20].

Given the limited evidence from South Asian populations for risk estimates for childhood obesity that are attributable to maternal diabetes in utero, further studies in these populations were identified as an important research need [7]. South Asians present with greater metabolic risk at lower levels of BMI compared with other ethnic groups, with type 2 diabetes developing at a younger age and rapidly progressing to other complications [21]. Being obese in childhood and adolescence is associated with obesity in the adult life, and overweight in adolescence is considered an important predictor of long-term morbidity and mortality [22–24]. Given the high risk of diabetes and cardiovascular diseases and rising trend of obesity among South Asians, it is imperative to identify factors that increase this risk.

This study sought to add to the limited evidence on long-term effects of HIP in South Asia by determining the association between the intrauterine exposure to hyperglycaemia and anthropometric measurements in offspring at 10-11 years of age in Sri Lanka.

## 2. Materials and Methods

### 2.1. Study Design and Population

A retrospective cohort study was conducted in eight Medical Officer of Health (MOH) areas in Colombo district, Sri Lanka, to assess the long-term outcomes of HIP on the mother and the offspring. We have previously published the risk of type 2 diabetes in the mothers 10 years after gestational diabetes [23].

Colombo is the most populous district in Sri Lanka with a total estimated population of 2,324,349 amounting to nearly 11% of the total population of the country [25]. For the delivery of public health services, the district is divided into fifteen MOH areas and the metropolitan Colombo Municipal Council area. Each MOH area is subdivided into Public Health Midwife (PHM) areas, which constitute the smallest field health care delivery unit in the public health system of Sri Lanka. The PHM delivers maternal and child care services as the grass root level health care worker and maintains a paper-based record keeping system for maternal and child care services. Live births in a given PHM area are recorded in the “Birth and Immunization Register” (BI Register) by the PHM. In the current study, we identified children born in 2005 through the BI registers and through schools in the selected MOH areas.

There was no universal screening programme to screen for HIP in Sri Lanka in 2005, and GDM screening in the antenatal clinics, as per national guidelines at that time, was based on assessment of risk factors [26]. These women underwent 75 g oral glucose tolerance testing mainly at gestation weeks 24–28. The World Health Organization (WHO) 1999 criteria for 2-hour post 75 g oral glucose load (≥140 mg/dl) was taken as the criterion for diagnosis of GDM [27].

Since Sri Lanka does not have an electronic database system for keeping patient records and paper-based records are stored only for 5 years in the health institutions, tracing patient-held antenatal records to verify the exposure status (hyperglycaemia in pregnancy) was the best possible option available. A feasibility study conducted beforehand to verify the availability of patient held antenatal records revealed that approximately 70% of women had antenatal records 10 years after the delivery.

The study was conducted in three stages. In the first stage of the study, a self-administered questionnaire to obtain information on history of hyperglycaemia in the index pregnancy, availability of antenatal records, and blood sugar assessment reports of the index pregnancy was sent to all mothers of 2005 born children identified through the BI registers in the community and through schools in the selected MOH areas. We defined occurrence of hyperglycaemia in the index pregnancy as a positive answer (yes) to the question “Did you have high blood sugar/diabetes during the index pregnancy”. Given the high literacy level among women in Sri Lanka, most women were aware of whether they had diabetes during pregnancy.

A total of 7205 children who were born in 2005 were identified in stage 1. The prevalence of self-reported hyperglyceamia in the index pregnancy was 3.5% (*N* = 257). Eighty-eight percent (*n* = 226) of mothers of children exposed to HIP still had antenatal records of index pregnancy compared with 69% (*n* = 4811) of mothers of children not exposed to HIP. Potential participants for the main study were identified at the end of the first stage. All children whose mothers had antenatal records and gave a history of HIP during the index pregnancy were considered as “potential participants” to be included in the “exposed group.” For each potential participant in the exposed group, two children of mothers with antenatal records and no history of HIP during the index pregnancy were selected from the same PHM area as “potential participants” to be included in the “nonexposed group.”

During the second stage, the mothers of all potential participants of “exposed” and “nonexposed” groups were invited to participate in the “eligibility assessment sessions.” These eligibility assessment sessions were conducted at PHM area level as it was easily accessible to all mothers, thus maximizing the participation. The research team interviewed the mothers of potential participants and scrutinized their antenatal and medical records to identify participants meeting the inclusion criteria (born in 2005, availability of antenatal records, and singleton pregnancy). Having received antenatal care in a unit lead by a consultant obstetrician was another inclusion criterion for both “exposed” and “nonexposed” groups to counter the possibility of misclassification due to limiting the GDM screening to high-risk pregnancies in 2005. So, mothers of all participants had received antenatal care from a consultant obstetrician-led team in a Tertiary care hospital, General hospital, or a Base hospital.

**Figure 1:**
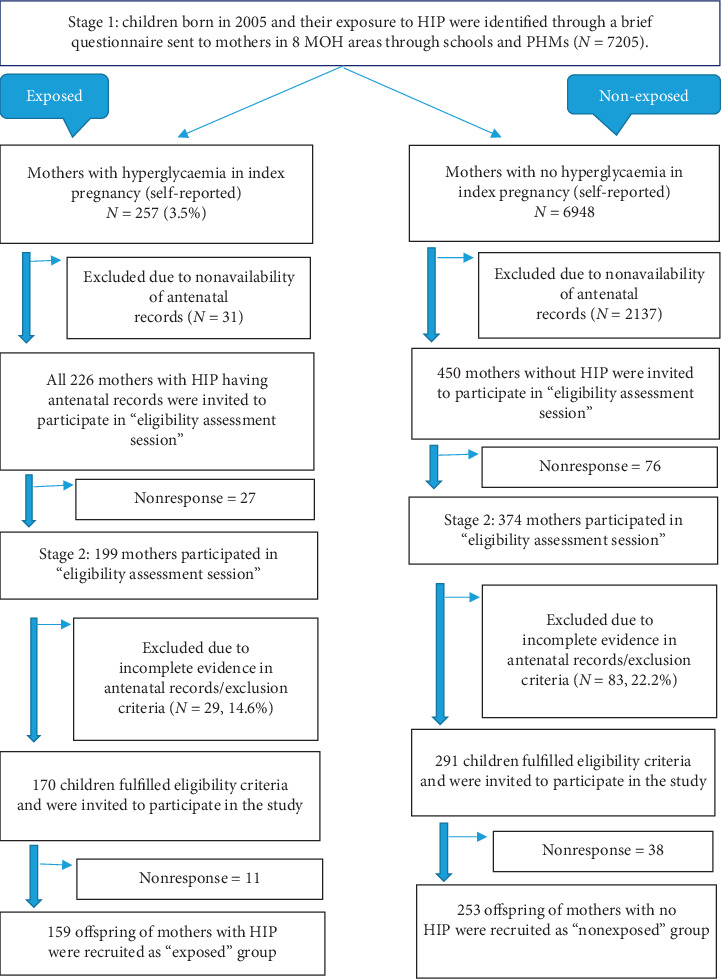
Selection of the study population.

One-hundred seventy children exposed to HIP and 291 children not exposed to HIP were identified as eligible and were invited for the study. A sample size of 161 in each group was required to detect a 15% difference in the risk of being overweight with 90% power, an alpha error of 0.05, and a 1 : 1 ratio between children exposed and not exposed to hyperglcyaemia in utero [28]. In the third stage, 159 offspring of women with HIP (OHIP) and 253 offspring of women with no HIP (ONHIP) in the index pregnancy participated in the study. Among the OHIP, 86.8% (*n* = 138) were exposed to gestational diabetes in utero. The detailed flow chart of participant selection is given in Figure 1.

### 2.2. Data Collection

Data collection was carried out by a team of doctors. “Data collection sessions” were arranged in a location easily accessible to participants in a given locality such as a field clinic centre or the MOH office. Sociodemographic characteristics and participants' physical activity engagement were obtained by interviewing the mothers. A 3-day diet diary and a 24-hour dietary recall were used to assess the participant's dietary energy intake. Energy intakes were calculated using the computerized food composition database, FoodBase 2000 software (Institute of Brain Chemistry, UK), containing Sri Lankan food items and mixed dishes [29] at the Department of Applied Nutrition, Wayamba University of Sri Lanka. Pregnancy-related information and glycaemic status during the index pregnancy were extracted from antenatal records to ascertain the exposure status using the WHO 1999 criteria for diagnosis of diabetes in pregnant women [27]. Maternal BMI in the first antenatal clinic visit in the first trimester was taken from the antenatal record as a proxy for the “maternal prepregnancy BMI”. According to national maternal care guidelines in Sri Lanka [26], BMI is measured and recorded as three categories (<18.5, 18.5–24.9, and ≥25) in the first clinic visit only if the woman presents before the completion of the 12^th^ week of gestation. As the data were extracted from the antenatal records, we had to limit to the above 3 categories of BMI when adjusting for maternal BMI.

Anthropometric measurements of the participants were obtained early in the morning following standard operating procedures to ascertain outcome status. Weight and height were measured in light clothing and without shoes. Weight was measured to the nearest 0.1 kg using a calibrated digital scale (SECA 876). Height was measured to the nearest 0.1 cm using a SECA stadiometer. Waist circumference was measured to the nearest 0.1 cm at the midpoint between the lowest rib and the top of the iliac crest with a nonelastic tape. Triceps skinfold thickness was measured to the nearest 0.2 mm using a Harpenden skinfold caliper. Two measurements were taken, and the mean was used for analysis. The same instruments were calibrated regularly and used throughout the study.

#### 2.2.1. Ascertainment of Exposure

Children with documentary evidence of exposure to HIP in antenatal records or glucose tolerance tests during the index pregnancy were classified as the OHIP (exposed) group. Diagnosis of GDM and diabetes mellitus in the mother was based on the WHO 1999 criteria [27] which was used in Sri Lanka in 2005. Children with no documented evidence of exposure to HIP in antenatal records during the index pregnancy were classified as the ONHIP (nonexposed) group.

#### 2.2.2. Ascertainment of Outcome

Anthropometric outcome measures were ascertained as follows.

We defined “overweight” and “obesity” with two different sets of reference BMI values: WHO growth standards [30] and International Obesity Task Force (IOTF) criteria [31, 32].

#### 2.2.3. Overweight

WHO standards: overweight was defined as age- and sex-adjusted BMI-for-age *z*-score > +1SD from the median [30].

IOTF criteria: overweight was defined as BMI greater than age and sex specific cutoffs for a projected BMI ≥25 kg/m^2^ at 18 years of age [32, 33].

#### 2.2.4. Obesity

WHO standards: obesity was defined as age- and sex-adjusted BMI-for-age *z*-score > +2SD from the median [30].

IOTF criteria: obesity was defined as BMI greater than age and sex specific cutoffs for a projected BMI ≥30 kg/m^2^ at 18 years of age [31, 32].

WHO AnthroPlus for personal computers software for assessing growth of the world's children and adolescents was used to calculate BMI and BMI-for-age *z*-scores [33].

#### 2.2.5. Abdominal Obesity

Abdominal (central) obesity was defined as waist circumference above the 90^th^ percentile for age and sex [34]. Since body fat distribution is different among children of Asian, African, and Caucasian races [35], WC percentiles developed for Indian children by Kurian et al. [36] were used to identify cutoff values to define abdominal obesity.

#### 2.2.6. High Triceps Skinfold Thickness

High triceps skinfold thickness was defined as TSFT above the 85^th^ percentile for age and sex. Since there are racial differences in skinfold thickness [37], triceps skinfold thickness reference charts developed for Indian children using the same instrument (Harpenden caliper) were used in this study [38].

### 2.3. Statistical Analysis

Baseline characteristics of participants in the OHIP and ONHIP groups were described using descriptive statistics. Variables were tested for normality using the Kolmogorov–Smirnov test. Normally distributed continuous data are presented as means (SD), and nonnormally distributed data are presented as medians (IQR). Frequencies and percentages were used to summarize categorical variables. Comparisons of baseline and follow-up assessment characteristics of OHIP and ONHIP groups were done using the *t* test (for normally distributed data) or Mann–Whitney *U* test (for nonnormally distributed data) for continuous variables and the chi-squared test for categorical variables. Unadjusted odds ratios and their 95% confidence intervals (CI) were calculated to assess the association between HIP and overweight, obesity, abdominal obesity, and high TSFT (Model 1). Binary logistic regression analysis was carried out to adjust for possible confounding effects of maternal prepregnancy BMI, maternal age at delivery, and parity (Model 2) and maternal prepregnancy BMI, maternal age at delivery, parity, and birth weight (Model 3). Based on the national guidelines on antenatal care in Sri Lanka, birth weight ≥3.5 kg was taken as macrosomia [26]. All tests of significance were two-tailed. A probability level of *p* < 0.05 was used to indicate statistical significance in all analyses.

### 2.4. Ethical Considerations

This study has been carried out in accordance with the Code of Ethics of the World Medical Association (Declaration of Helsinki) for experiments involving humans. The protocol was approved by the Ethics Review Committee of the Faculty of Medicine, University of Kelaniya, Sri Lanka (Ref. No. P/24/03/2015). All mothers of study participants gave informed written consent, and verbal assent was obtained from the participants. A “feedback session” was arranged after each data collection session, and participants were issued a personal record with anthropometric measurements. Participants needing specialized care were referred to the Lady Ridgeway Children's Hospital, a tertiary care facility for children, in Colombo.

## 3. Results

### 3.1. Characteristics of the Study Population

A total of 412 children born in 2005 participated in the study. Baseline characteristics of the 159 offspring of women with HIP (OHIP) and 253 offspring of women with no HIP (ONHIP) are compared in Table 1. Eighty seven percent (*n* = 138) of the women with HIP had gestational diabetes while others (*n* = 21 or thirteen percent) had diabetes in pregnancy (DIP).

At the time of the outcome assessment, the age of all participants ranged between 10.3 years and 11.6 years with a mean of 10.85 years (SD = 0.39). Mothers of children exposed to HIP were older and had significantly higher BMI at the booking visit in the first trimester compared with mothers of nonexposed children (*p* < 0.001). Exposed children were heavier at birth and had a shorter gestational age compared with nonexposed children (*p* < 0.001). About half of the children in ONHIP group were firstborns compared with only one-third of children in the OHIP group (*p*=0.002). Sociodemographic characteristics, breast-feeding practices, dietary energy intake, and physical activity level were not significantly different between the two groups.

### 3.2. Outcome Assessment

Table 2 compares the summary statistics of anthropometric measurements between OHIP and ONHIP groups.

Anthropometric measurements were assessed for normality using the one-sample Kolmogorov–Smirnov test. Height and BMI-for-age *z*-score were normally distributed while weight, BMI, WC, and TSFT were not normally distributed.

The mean BMI-for-age *z*-score of exposed children was significantly higher than that of nonexposed children (*p* < 0.001). Exposed children were significantly heavier and had significantly higher median BMI, WC, and TSFT than the nonexposed children (*p* < 0.001).

Table 3 shows the anthropometric outcome status of children at follow-up.

Prevalence of overweight and obesity by WHO standards was higher than the prevalence determined by the IOTF criteria. The prevalence of overweight, abdominal obesity, and high TSFT was significantly higher among the offspring exposed to HIP. Children exposed to HIP had 2 times the odds of developing overweight and abdominal obesity and have a TSFT ≥85^th^ percentile than the nonexposed children (*p* < 0.01).

### 3.3. Association between HIP and Anthropometric Outcome Measures after Adjusting for Confounders

Logistic regression analysis was carried out to describe the association between the HIP and anthropometric outcome status (overweight, abdominal obesity, and TSFT ≥ 85^th^ percentile) in 10- to 11-year-old children after adjusting for maternal BMI in the first trimester, parity of index pregnancy, maternal age at delivery, and birth weight (Table 4).

Even after adjusting for maternal BMI, maternal age, birth weight, and birth order, exposure to HIP was a significant predictor of overweight and abdominal obesity in the offspring at 10 years of age in both Models 2 and 3. HIP was a significant predictor of TSFT ≥ 85^th^ percentile in Model 2, but after adjustment for birth weight in Model 3, the association between HIP and TSFT ≥ 85^th^ percentile attenuated towards the null. A sensitivity analysis confined to offspring of GDM did not reveal the differences in the outcome measures (data not shown).

Maternal overweight in the first trimester, a proxy for prepregnancy overweight, is an independent risk factor for offspring overweight and high TSFT at 10-11 years. Similarly, being the first-born child carries a more than two-fold increased risk of overweight independent of maternal BMI, birth weight, and exposure to HIP.

## 4. Discussion

To the best of our knowledge, this is the first study on long-term implications of HIP on anthropometric parameters in the offspring in Sri Lanka and one among the handful of studies from South Asia. Even the previous studies conducted in India [39, 40] were limited by the small number of offspring of GDM mothers (*n* = 41 and *n* = 35). The significant associations between maternal HIP and overweight, abdominal obesity, and high TSFT in the offspring in this study support the hypothesis that intrauterine exposure to HIP may have a long-term risk of increased adiposity in the offspring. The higher BMI and BMI-*z*-score in the offspring of women with HIP reported in this study are consistent with those of the earlier studies [10–12, 14, 18, 20, 41–45]. A comprehensive meta-analysis by Philipps et al. identified a strong association between intrauterine exposure to maternal diabetes and increased offspring BMI in childhood [46]. Use of two widely used BMI reference standards to define overweight and obesity in the present study allowed comparison with other studies. Overweight and obesity prevalence in the exposed group reported in the HAPO FUS study is much higher than the prevalence observed in our study (39.5% and 19.1% vs 25.8% and 3.8%, respectively) using the IOTF criteria [20]. However, the effect size for the risk of overweight was larger in the present study (unadjusted OR = 2.4 in the present study compared with 1.5 in the HAPO FUS study) [20]. In the present study, prevalence of overweight (BMI-*z*-score > +1SD) was significantly higher among OHIP compared with ONHIP (30.8% vs 16.2% by the WHO growth standards). Our results are similar to findings of other studies that have reported a higher risk of overweight and obesity among offspring of mothers who had HIP [9, 14, 20, 42, 47, 48]. However, in contrast to other studies, the prevalence of obesity (BMI-*z*-score > +2SD) was similar in the exposed and nonexposed groups in our study.

We observed that children exposed to intrauterine hyperglycaemia had a significantly higher waist circumference at 10 years compared with nonexposed children. Previous studies have reported similar findings of significantly higher waist circumference among offspring exposed to hyperglycaemia in utero including a multinational study involving 206 offspring of GDM mothers and 4534 offspring of non-GDM mothers from 12 countries [15, 20, 49, 50].

In our study, children exposed to HIP had significantly higher TSFT than children not exposed to HIP (13.3 mm vs 9.9 mm; *p* < 0.001) in unadjusted analysis and Model 2. In addition, the significantly higher TFST in children with a higher birth weight even after adjusting for exposure to HIP suggesting an additional risk of birth weight has been previously reported by some authors [51]. Wright et al. observed that children exposed to GDM had significantly higher sum of skinfold thicknesses (subscapular and triceps) than that of nonexposed children [52]. Crume et al reported an increased subscapular to triceps skinfold thickness ratio in children exposed to HIP [50]. Krishnaveni et al. from India observed significantly higher TSFT among the offspring of diabetic mothers compared with offspring of nondiabetic mothers at 5 years of age [39]. When the same cohort was assessed at 9.5 years of age, they observed a significantly higher BMI and TSFT among girls exposed to intrauterine hyperglycaemia but not among boys [42]. No significant difference between the growth of the boys and girls was observed in our study (results not shown).

In contrast to the many studies where the association between maternal HIP and child overweight attenuated towards the null after adjusting for maternal BMI [41, 47, 50, 53], our results were statistically significant even after adjusting for maternal BMI, maternal age, child's birth weight, and birth order.

We included offspring of women with any type of HIP (gestational diabetes, pre-existing diabetes, or overt diabetes first detected in pregnancy) in the “exposed” group without stratification by type of diabetes based on the previous research which showed that long-term consequences of HIP on offspring overweight are independent of mother's diabetes type [8, 54, 55]. A subgroup analysis of a meta-analysis by Philips et al revealed that there is no difference in offspring BMI-*z*-score in relation to diabetes types such as GDM or Type 1 diabetes [46].

Using three methods (BMI, waist circumference and triceps skinfold thickness) as measures of adiposity is a unique strength of this study. BMI is widely used to measure body composition and is used to define overweight and obesity [56]. Though BMI is widely used to measure generalized obesity or adiposity, its value in discriminating lean body mass from fat mass has been challenged [57]. Skinfold thickness is a valid measurement of subcutaneous fat [58], and there is evidence to suggest that later adulthood adiposity is better predicted by adolescent skinfold thickness than by adolescent BMI [59]. In predicting cardiovascular disease risk, abdominal adiposity appears to be superior to BMI [56]. Abdominal obesity, defined as waist circumference >90^th^ percentile, is a mandatory criterion for diagnosing metabolic syndrome in children and adolescents [34]. Since body fat distribution is different among children of Asian, African, and Caucasian races [35], waist circumference percentiles developed for Indian children based on measurements made on 9060 children 3–16 years of age [36] were used to identify cutoff values to define abdominal obesity in the present study. Similarly, triceps skinfold thickness reference charts developed for Indian children were used in this study [38].

Having a large number of offspring exposed to HIP is a major strength of our study. Selecting both “exposed” and “nonexposed” children from the same source population in the community based on antenatal records reduced recall bias and misclassification. Since exposure was assigned on an earlier date than the outcome was measured in the child, it is unlikely that the outcomes of interest would have influenced the classification of exposure status. Children whose mothers received antenatal care from a consultant obstetrician were selected in both exposed and nonexposed groups. Since universal screening for HIP was not available in 2005, having being under the care of a consultant obstetrician implies that they had a fair chance of being screened and diagnosed for HIP, if required, thus minimizing misclassification bias. Even if misclassification did occur, the associations between HIP and anthropometric outcome measures we observed are likely to be an underestimation. We compared the prevalence of overweight and obesity in exposed and nonexposed children calculated with two different sets of reference BMI values: WHO growth standards and the IOTF criteria to provide more opportunities of comparison between studies.

This study has several limitations. Not having detailed information on maternal blood sugar levels at diagnosis and glycaemic control during pregnancy is a limitation of this study.

Missing maternal prepregnancy BMI data on nearly 30% of mothers is another limitation. It is likely that some of these women whose BMI data were not available presented for the booking visit after 12 weeks of gestation. It would have been ideal if we adjusted for the weight gain in pregnancy. But these data were not available for the majority of the participants. We adjusted for the birth weight of the child which can be taken as a proxy measure for weight gain in pregnancy.

The results of this study have several important public health implications. This study adds to the limited evidence currently available on this subject from South Asia. Locally generated evidence in this study would be an eye opener for clinicians, field health care workers, and health policy-makers to take necessary actions to follow up the exposed children closely during the critical period of development to prevent and to detect the appearance of anthropometric risk parameters early. Creating awareness on possible long-term effects of maternal hyperglycaemia would motivate women to achieve better glycaemic control during pregnancy and lifestyle modification of the child with adherence to a healthy diet and increased physical activity to reduce the risk of overweight. Given the high prevalence of HIP in Sri Lanka and other South Asian countries, preventive strategies targeted at women of childbearing age and offspring of women with HIP are likely to have a significant population health impact on the current epidemic of obesity and noncommunicable diseases.

## 5. Conclusions

Children exposed to intrauterine hyperglycaemia have a higher risk of overweight and abdominal obesity at 10-11 years independent of maternal prepregnancy overweight, maternal age, birth weight, and birth order. The findings of this study add to the limited body of knowledge regarding long-term effects of HIP on the offspring in South Asian populations.

## Figures and Tables

**Table 1:** Characteristics of the exposed (OHIP) and nonexposed (ONHIP) groups.

Characteristic	OHIP group (*N* = 159)	ONHIP group (*N* = 253)	*p* value
*Sociodemographic characteristics*			
Age years, mean (SD)	10.89 (0.32)	10.82 (0.31)	0.009^a^
Sex, male	*N* = 67 (42.1%)	*N* = 118 (46.6%)	0.37^b^
Ethnicity, Sinhala	*N* = 153 (96.2%)	*N* = 231 (91.3%)	0.24^b^
Education level of mother			
Primary education	*N* = 5 (3.1%)	*N* = 2 (0.8%)	0.10^b^
Secondary education	*N* = 144 (90.6%)	*N* = 241 (95.2%)	
Tertiary education and higher	*N* = 10 (6.3%)	*N* = 10 (4.0%)	
Family income per month			
<LKR 50000 (<USD 265)	*N* = 113 (71.1%)	*N* = 190 (75.1%)	0.37^b^

*Index pregnancy-related characteristics*			
Mother's age at delivery in years, mean (SD)	31.9 (5.3)	27.8 (5.3)	<0.001^a^
Primi parity	*N* = 53 (33.3%)	*N* = 128 (50.6%)	0.002^b^
Mother's BMI at first trimester of index pregnancy^1^			
<18.5	*N* = 5 (5%)	*N* = 43 (22.6%)	<0.001^b^
18.5–24.9	*N* = 56 (55.4%)	*N* = 131 (69.0%)	
≥25	*N* = 40 (39.6%)	*N* = 16 (8.4%)	
Gestational age in weeks, median (IQR)	38 [38–40]	40 [37–39]	<0.001^c^
Gestational age at delivery ≥37 weeks	*N* = 145 (91.2%)	*N* = 246 (97.2%)	0.007^b^
Birth weight of index child in kg, mean (SD)	3.1 (0.5)	2.9 (0.4)	<0.001^a^
Birth weight of index child ≥3.5 kg	*N* = 39 (24.5%)	*N* = 20 (7.9%)	<0.001^b^
Exclusive breast-feeding duration ≥4 months	*N* = 141 (88.7%)	*N* = 236 (93.2%)	0.10^b^

*Lifestyle-related characteristics*			
Physical activity (>1 hour/d for ≥5 days/week)^2^	*N* = 71 (44.7%)	*N* = 130 (51.6%)	0.17^b^
Dietary energy intake in kCal, median (IQR)^3^	1449 (1238–1864)	1514 (1257–1850)	0.78^c^

^1^Data were available in 63.5% (*N* = 101) of OHIP and 75.1% (*N* = 190) of ONHIP. ^2^Data available for 159 OHIP and 252 ONHIP. ^3^Data available for 70.4% (*N* = 112) OHIP and 74.3% (*N* = 188) ONHIP. ^a^Independent sample *T* test. ^b^Chi-squared test. ^c^Mann–Whitney *U* test.

**Table 2:** Anthropometric assessment at follow-up.

Characteristic	OHIP group (*N* = 159)	ONHIP group (*N* = 253)	*p* value
Height (cm), Mean (SD)	141.5 (6.9)	140.4 (6.9)	0.09^a^
Weight (kg), median (IQR)	34.1 (28.3–41.6)	28.8 (25.6–36.5)	*p* < 0.001^b^
BMI, median (IQR)	16.9 (14.7–20.2)	15.1 (13.8–17.8)	*p* < 0.001^b^
BMI *z-*score, Mean (SD)	−0.1 (1.6)	−0.9 (1.7)	*p* < 0.001^a^
WC (cm), median (IQR)	61.4 (55.7–69.2)	56.4 (52.7–64.4)	*p* < 0.001^b^
TSFT (mm)^1^, median (IQR)	13.3 (9.6–17.2)	9.9 (7.4–14.1)	*p* < 0.001^b^

BMI = body mass index. WC = waist circumference. TSFT = triceps skinfold thickness. ^1^Data available for 152 exposed and 236 nonexposed children. ^a^Independent samples *T* test. ^b^Mann–Whitney *U* test.

**Table 3:** Outcome status of participants at follow-up.

Outcome status	OHIP group (*N* = 159)	ONHIP group (*N* = 253)	Odds ratio (95% CI of OR)	*p* value
	Prevalence (95% CI)	Prevalence (95% CI)		
Overweight by WHO^a^ criteria (BMI^b^*z*-score > +1SD)	30.8% (23.6–37.9)	16.2% (11.6–20.7)	2.3 (1.4–3.7)	*p* < 0.001
Overweight by IOTF^c^ criteria	25.8% (18.9–32.7)	12.6% (8.5–16.7)	2.4 (1.4–4.0)	*p*=0.001
Obesity by WHO^a^ criteria (BMI^a^ z-score > +2SD)	5.7% (3.0–10.4)	5.1% (3.0–8.6)	1.1 (0.4–2.6)	*p*=0.82
Obesity by IOTF^c^ criteria	3.8% (0.8–6.8)	3.2%(1.0–5.4)	1.2 (0.4–3.5)	*p*=0.7
Abdominal obesity (WC^d^ ≥ 90^th^ percentile)	15.1% (3.9–10.2)	2.3 (1.2–4.4)	*p*=0.009
TSFT^e^ ≥ 85^th^ percentile	21.1 (14.5–27.7)	8.9% (5.2–12.6)	2.7 (1.5–4.9)	*p*=0.001

^a^WHO = The World Health Organization. ^b^BMI = body mass index. ^c^IOTF = International Obesity Task Force. ^d^WC = waist circumference. ^e^TSFT = triceps skinfold thickness.

**Table 4:** Predictors of anthropometric outcome status.

Risk factor	Overweight by WHO criteria (BMI *z* score > +1SD)			Overweight by IOTF criteria			Abdominal obesity (waist circumference ≥ 90^th^ percentile)			TSFT ≥ 85^th^ percentile		
	Model 1 (unadjusted) OR (95% CI)	Model 2 OR (95% CI)	Model 3 OR (95% CI)	Model 1 (unadjusted) OR (95% CI)	Model 2 OR (95% CI)	Model 3 OR (95% CI)	Model 1 (unadjusted) OR (95% CI)	Model 2 OR (95% CI)	Model 3 OR (95% CI)	Model 1 (unadjusted) OR (95% CI)	Model 2 OR (95% CI)	Model 3 OR (95% CI)
Exposure to HIP	2.3^*∗∗*^ (1.4–3.7)	2.6^*∗*^ (1.4–4.9)	2.5∗ (1.3–4.7)	2.4^*∗∗*^ (1.4–4.0)	2.5^*∗*^ (1.3–4.9)	2.3^*∗*^ (1.2–4.6)	2.3^*∗*^ (1.2–4.4)	2.7^*∗*^ (1.1–6.5)	2.9^*∗*^ (1.2–6.9)	2.7^*∗∗*^ (1.5–4.9)	2.2^*∗*^ (1.06–4.7)	1.9 (0.9–4.2)
Maternal BMI ≥ 25 kg/m^2^ in the first trimester	3.3^*∗∗*^ (1.8–6.2)	2.8^*∗*^ (1.4–5.8)	2.8^*∗*^ (1.4–5.8)	3.8^*∗∗*^ (2.0–7.3)	3.1^*∗*^ (1.5–6.5)	3.1^*∗*^ (1.5–6.5)	2.4^*∗*^ (1.1–5.6)	1.8 (0.7–4.6)	1.9 (0.7–4.7)	3.1^*∗*^ (1.5–6.3)	2.2 (0.9–4.8)	2.1^*∗*^ (1.01–5.1)
Firstborn child	1.6^*∗*^ (1.01–2.6)	2.7^*∗*^ (1.4–5.1)	2.7^*∗*^ (1.4–5.1)	1.5 (0.9–2.5)	2.2^*∗*^ (1.1–4.4)	2.2^*∗*^ (1.1–4.4)	1.4 (0.7–2.7)	1.8 (0.8–4.2)	1.8 (0.8–4.3)	1.1 (0.6–1.9)	1.6 (0.8–3.3)	1.6 (0.8–3.3)
Maternal age at delivery ≥35 years	0.8 (0.5–1.5)	1.1 (0.5–2.6)	1.1 (0.5–2.4)	1.1 (0.6–2.1)	1.0 (0.4–2.5)	0.9 (0.7–2.4)	0.7 (0.3–1.6)	0.9 (0.3–2.9)	1.0 (0.3–3.1)	3.4 (1.03–11.4)	2.8 (0.8–10.3)	2.4 (0.6–8.7)
Birth weight ≥ 3.5 kg	1.9^*∗*^ (1.03–3.4)	—	1.6 (0.7–3.5)	1.9^*∗*^ (1.01–3.7)	—	1.9 (0.9–4.2)	0.7 (0.2–2.1)	—	0.7 (0.2–2.1)	2.1^*∗*^ (1.04–4.2)	—	2.4^*∗*^ (1.05–5.3)

HIP = hyperglycaemia in pregnancy. TSFT = triceps skinfold thickness. BMI = body mass index. Model 1, unadjusted. Model 2, adjusted for exposure to HIP, maternal BMI, parity, and maternal age at delivery. Model 3, adjusted for exposure to HIP, maternal BMI, parity, maternal age at delivery, and birth weight. ^*∗*^*p* < 0.05. ^*∗∗*^*p* < 0.001.

## Data Availability

The dataset used to support the findings of this study is available within the article.
